# Various Adjuvants Effect on Immunogenicity of Puumala Virus Vaccine

**DOI:** 10.3389/fcimb.2020.545371

**Published:** 2020-10-26

**Authors:** Svetlana S. Kurashova, Aidar A. Ishmukhametov, Tamara K. Dzagurova, Maria S. Egorova, Maria V. Balovneva, Nikolai A. Nikitin, Ekaterina A. Evtushenko, Olga V. Karpova, Anna A. Markina, Peter G. Aparin, Petr E. Tkachenko, Vyatcheslav L. L`vov, Evgeniy A. Tkachenko

**Affiliations:** ^1^ Chumakov Federal Scientific Center for Research and Development of Immune-and-Biological Products of Russian Academy of Sciences, Moscow, Russia; ^2^ Institute for Translatonal Medicine and Bionechnology, Sechenov First Moscow State Medical University, Moscow, Russia; ^3^ Department of Virology, Lomonosov Moscow State University, Moscow, Russia; ^4^ National Research Center – Institute of Immunology Federal Medical-Biological Agency of Russia, Moscow, Russia; ^5^ Department of Internal Medicine Propaedeutics, Sechenov First Moscow State Medical University, Moscow, Russia

**Keywords:** ****hantavirus, hemorrhagic fever with renal syndrome, vaccine, adjuvants, immune response, heat-labile enterotoxin, low endotoxic lipopolysaccharide, plant virus-based spherical particles

## Abstract

Various adjuvant effects on the immunogenicity of the candidate inactivated Puumala virus vaccine were detected in BALB/c mice. Adjuvants under study were: aluminum hydroxide, spherical particles of Tobacco mosaic virus coat protein, B subunit of heat-labile enterotoxin of *Escherichia coli*, and low endotoxic lipopolysaccharide of *Shigella sonnei*. Aluminum hydroxide (1 mg/ml) did not affect neutralizing antibodies’ induction and vaccine stability during storage compared to immunization with the vaccine without adjuvant. B subunit of heat-labile enterotoxin (0.2 µg/ml), low endotoxic lipopolysaccharide (50 µg/ml), and plant virus-based spherical particles (300 µg/ml) significantly enhance the humoral immune response of vaccine (p < 0.0001). Pronounced stimulation of IL-12 and IFN-*ɣ* was observed when mice were immunized with vaccines both with adjuvants (except of aluminum hydroxide) and without adjuvants. It has been shown that low endotoxic lipopolysaccharide contributes not only to enhance the immune response but also to stabilize vaccine immunogenicity during at least 1 year storage.

## Introduction

Hemorrhagic fever with renal syndrome (HFRS) is rodent-borne viral zoonoses caused by the viruses of family *Orthohantavirus*, order *Bunyavirales*. The disease is widespread in Eurasia, and in Russia occupies a leading position among all natural focal human diseases. More than 97% of HFRS cases in the Russian Federation are caused by the Puumala virus (PUUV), and about 3% of cases are caused by the Hantaan, Amur, Seoul, Kurkino, and Sochi viruses (Tkachenko et al., 2016a; Tkachenko et al., 2016b; Tkachenko et al., 2019). Monovalent and bivalent inactivated vaccines based on the epidemiologically significant strains of the Puumala and Kurkino viruses were developed in Russia (Barkhaleva et al., 2011; Tkachenko et al., 2015), and polyvalent vaccine based on the Puumala, Hantaan and Sochi viruses, which successfully passed preclinical tests (Sinyugina et al., 2019).

It is known that the disadvantage of inactivated vaccines, compared with live ones, is an insufficiently intense or prolonged immune response, which implies revaccination after a certain period. To boost immunogenicity inactivated vaccines need to be supplemented with adjuvants. Adjuvants represent a heterogeneous group of compounds in their chemical composition and mechanism of action (Ciabattini et al., 2016). The adjuvant immunostimulation effect may be due to immunomodulation, optimization of antigen presentation, induction of the T-cells, targeting and depot formation (McKee et al., 2009; McKee and Marrack, 2017). Only aluminum hydroxide was used as adjuvant in the previously described brain-derived (Krüger et al., 2001; Choi et al., 2003) and cultural (Cho and Howard, 1999; Hooper and Li, 2001; Tkachenko et al., 2009; Tkachenko et al., 2015) vaccines against HFRS. It is assumed that the inclusion of adjuvants in vaccines provides a reliable immune response with fewer immunizations (Halperin et al., 2006; Banzhoff et al., 2009; Schwarz et al., 2009), increases the tension and duration of immunity (Galli et al., 2009), and harmonizes the Th1 and Th2 immune responses (Khurana et al., 2010).

Aluminum hydroxide (Al) represents a class of mineral adjuvants and has been traditionally included in inactivated vaccines (Savelkoul et al., 2015). In humans, as a rule, proteins adsorbed on Al stimulate Th2 and Th1 cell response (Didierlaurent et al., 2009). However, as it was shown in experiments on mice, Al is a relatively poor immunostimulant, especially in inducing a cellular immune response by means of inhibiting IL-12 secretion by dendritic cells (Mori et al., 2012; McKee and Marrack, 2017) and induces a deeply polarized Th2 cell response (Aguilar and Rodriguez, 2007; Li et al., 2007; Kool et al., 2011). Thus, the main disadvantages of aluminum adjuvants are: lack of exposure to Th1 immunity, increased production of IgE, potential neurotoxicity, nephrotoxicity (McKee and Marrack, 2017), inflammation (granuloma) in the injection area (Kozlov et al., 2014).

Another group of interest is corpuscular adjuvants. This group of adjuvants includes liposomes, polymer micro particles, nanoparticles, virus-like particles, and immunostimulating complexes (Bonam et al., 2017). Among them, plant viruses and their virus-like particles are of particular interest and are being intensively explored at present. Plant viruses feature as the complete biosafety for mammals and humans, and immunogenicity makes them an attractive tool for vaccine development (Balke and Zeltins, 2019). Here the spherical particles (SPs) obtained by thermal remodeling of helical rod-shaped Tobacco mosaic virus were used. The unique feature of SPs is their ability to non-specifically adsorb foreign proteins and even non-enveloped virions on the surface (Karpova et al., 2012; Trifonova et al., 2014). Furthermore, SPs can enhance the immune response to the target antigen and act as an adjuvant (Trifonova et al., 2017). In addition, the absence of toxic effects of SPs for laboratory animals was shown (Nikitin et al., 2018a; Nikitin et al., 2018b).

Also of great interest are protein adjuvants, such as the heat-labile enterotoxins (LTs) *Vibrio cholerae* (Holmes et al., 1995; Pizza et al., 2001) and *Escherichia coli* (Wang H. et al., 2019). LTs are used as a molecular carrier in a bivalent vaccine for the prevention of brucellosis and diarrhea caused by *Brucella abortus* and enteropathogenic *E. coli*, *Vibrio cholera* in veterinary^1^. The LTB of *E. coli* can be used as an adjuvant. In this case, IgG and IgA are produced against the targeting antigens (Clements et al., 1988). The mechanism of this phenomenon is not completely clear, but it has been shown that in addition to the humoral immune response, the Th1 pathway is stimulated (Sasaki et al., 2000). It is assumed that LTB can induce functional activation of bone marrow dendritic cells and stimulate CD4+ T-cell proliferation by producing cytokines and increasing the co-stimulating molecules necessary for effective activation of T-cells (Spörri and e Sousa, 2005; Liang et al., 2009). It is also possible to mediate immunostimulation *via* Toll-receptors 2 (TLR2), which can lead to the regulatory dendritic cell generation and T-cell induction (Dillon et al., 2006). The adjuvant effect of LTB is enhanced when combined with antigens that are effectively represented by macrophages and dendritic cells (George-Chandy et al., 2001). LTB can stimulate both mucosal and systemic immune responses (Scharek and Tedin, 2007).

Lipopolysaccharide (LPS) is a powerful Th1 adjuvant (McAleer and Vella, 2008), which has a multifactorial mechanism for controlling the immune response, including induction of IL-12 and IFN-*γ* interleukin, T-cell survival factors and DC activation through type I IFN (Longhi et al., 2009; McAleer and Vella, 2010). LPS also stimulates cytotoxic T-cell response, which is poorly obtained by standard (Th2) adjuvants and can be effective against intracellular pathogens (Gregg et al., 2017). However, the use of LPS as a vaccine component was limited due to its high endotoxicity. We use as adjuvant novel clinically applicable apyrogenic low-endotoxic LPS from *Shigella sonnei*. The native LPS was purified from *S. sonnei* by the Westphal method (Westphal, 1965) and then fractionated by Sephadex G-150 gel-permeation chromatography in the presence of Na-deoxycholate to give lipopolysaccharides with a long chain O-specific polysaccharide—S-LPS. S-LPS were partially deacylated under alkaline conditions to give LPS with mainly a tri-acylated lipid A moiety—Ac_3_-S-LPS, without admixtures of penta- and hexa-acylated lipid A. The structural analog of Ac_3_-S-LPS from *Shigella flexneri* 2a successfully passed clinical trials as candidate vaccine against *S. flexneri* 2a infection (Ledov et al., 2019).

The aim of the study was to evaluate the effect of various origin adjuvants on the immunogenicity of the inactivated Puumala virus vaccine.

## Materials and Methods

### Viruses and Cells

Candidate Puumala vaccine (hereinafter VAC) was developed on the basis of strain PUU-TKD/VERO (GenBank accession number: BankIt 2108429: S-MH251331; M-MH251332; L-MH251333), propagated in the Vero cells (WHO Vero cell bank ECACC, Accession number 991042) according to previously developed technology (Tkachenko et al., 2015). Briefly, Vero cells were cultured in Eagle’s medium (EMEM) supplemented with 10% calf blood serum, gentamicin sulfate (50 μg/ml). The cell monolayer was infected in 1 L roller flasks with a multiplicity of infection (MOI) 0.01–0.05. EMEM medium without calf serum was used for virus growth. Virus yields (infected culture supernate) were collected daily from 5 to 9 days. The culture supernate was inactivated with 98% beta-propiolactone (AcrosOrganics, Geel, Belgium) in final dilution 1/6,000. Completeness of virus inactivation was assessed in three consecutive passages of the candidate killed vaccine through the Vero cell culture followed by virus indication by means of IFA and FFU. Virus was concentrated by centrifugation in the tangential flow following clarifying filtration followed by purification on the Sepharose 6FF and Capto Сore 700 sorbents by means AKTA purifier chromatograph and C series columns (GE Healthcare).

VAC dose was calculated according to the number of PUUV RNA copies/ml that can induce NAb in BALB/c mice in titer ≥4,32 log_2_ during vaccine storage for 1 year. In our preliminary experiments, such a dose for the beta-propiolactone-inactivated PUUV vaccine should be ≥5 × 10^4^ RNA copies/ml.

One dose (1.0 ml) of the VAC contains: inactivated Puumala virus—6.91 × 10^4^ copies/ml, total protein—38 µg/ml (by Lowry’s method); cellular DNA <10 ng/ml (by RT-PCR), phosphate buffered salt solution (PBS)*—solvent.

*PBS solution: sodium chloride (Eur.Ph.) —4 mg; potassium chloride (Eur.Ph.) —0.1 mg, sodium phosphate (Eur.Ph.) —0.71 mg; potassium phosphate (Eur.Ph.) —0.12 mg.

### Adjuvants

1) Aluminum hydroxide [Alhydrogel^®^ “85” 2% (Brenntag GmbH, German) with approximately 20% higher protein adsorption capacity] was applied in concentration of 1 mg/ml. 2) Spherical particles (SPs), which were generated by heating of Tobacco mosaic virus at 98°C (Karpova et al., 2012; Trifonova et al., 2015). The average particle size was 260 nm. We studied the adjuvant effect of SPs in the amount of 100, 150, and 300 μg per 1 ml of vaccine. 3) Heat-labile enterotoxin B (LTB) is a recombinant enterotoxigenic *Escherichia coli* protein purified by affinity chromatography^1^. LTB was used in an amount of 7.5 and 0.2 μg/ml. 4) LPS, a homogenous *S. sonnei* LPS subfraction—Ac_3_-S-LPS, containing long chain O-specific polysaccharide (S-LPS) and mainly tri-acylated lipid A moiety without admixtures of penta- and hexa-acylated lipid A (Ledov et al., 2019). LPS was applied in a dose of 50 μg/ml.

Undiluted and diluted vaccines were added the same amount of the adjuvants.

### Immunization

Specific-pathogen-free, 6-week-old female BALB/c mice obtained from the Andreevka branch of the Federal State Institution of Science “Scientific Center for Biomedical Technologies of the Federal Medical and Biological Agency” of Russia. All mice were kept in accordance with the laboratory animal control guidelines and were housed in the same conditions of maintenance and feeding in the facility of the Chumakov Federal Scientific Center for Research and Development of Immune-and- Biological Products of Russian Academy of Sciences.

Mice were randomly assigned to groups (seven mice per group). Experimental vaccines were intramuscularly (i/m) injected with 500 μl (0.5 dose of vaccine) and were boosted on day 14 post-primary and second immunizations. Mice from the control (C) group were intramuscularly injected with 500 μl PBS solution with corresponding adjuvants. The experimental vaccines were administered intramuscularly undiluted (u/d) and in dilutions of ^1^/_2_, ^1^/_4_, and ^1^/_8_. Mice were bled by retro-orbital plexus puncture the day prior to the first injection for pre-immune reference sera (PI) for negative control. For immune response study mice were bled on day 14 after second and third immunizations. Animals were exsanguinated by placement in a CO_2_ chamber followed by bleeding *via* decapitation.

The collected sera were aliquoted. The vast majority of studies were done with blood sera stored at 6 ± 2°C for no more than a week. Part of serum aliquots were frozen and stored at −80°C for no more than a month, thawed on ice, and then tested within 24 h.

Experimental groups: 1—VAC; 2—VAC-AL (1 mg/ml); 3—VAC-SP/100 (100 μg/ml); 4—VAC-SP/150 (150 μg/ml); 5—VAC-SP/300 (300 μg/ml); 6—VAC-LTB/0.2 (0.2 μg/ml); 7—VAC-LTB/7.5 (7.5 μg/ml); 8—VAC-LPS (50 μg/ml).

Control groups: PI-(intact mice); C-PBS; C-AL; C-SP/100; C-SP/300; C-LTB/0.2; C-LTB/7.5; C-LPS (in these groups, the vaccine was replaced by PBS).

### Ethics Statement

Animal studies were carried out in accordance with the ethical principles established by the European Convention for the Protection of Vertebrate Animals used for experimental and other scientific purposes approved in Strasbourg on 03/18/1986 and confirmed in Strasbourg on 06/15/2006. The research Protocol was approved by the Ethics Committee of the Chumakov Federal Scientific Center for Research and Development of Immune-and-Biological Products of Russian Academy of Sciences. No deterioration in the state of animal health was detected during the experiments. Biohazard experiments, including live viruses and cells, were conducted in a laboratory under Biosafety Level 3 (BSL-3).

### Evaluation of Humoral Immune Response

Neutralizing antibodies titer (NAb) was determined by the focus-reduction neutralization test (FRNT_50_) using Vero E6 cells (ATCC^®^ No. CRL-1586) as previously described (Tkachenko et al., 2005). Briefly, serum samples were diluted twofold steps, mixed with an equal volume of the virus containing 50–100 focus-forming units and incubated for 1 h at 37°C for adsorption of non-neutralized virus. In control wells test virus was incubated with Eagle’s MEM alone. For the negative control sera of non-immunized mice were used; for the positive control HFRS convalescent sera were applied. Virus–serum mixtures were inoculated onto the confluent Vero E6 cells. After 6–7 days of incubation, ELISA was used to detect the focus forming units (FFU). Neutralizing antibodies titer was expressed as the reciprocal of highest serum dilution that resulted in greater than 50% reduction in the FFU number relative to the average FFU number in the virus control wells.

Each serum sample was tested three times in FRNT_50_. In the control mice group (C) the NAb titer did not exceed 2.32 log_2_ (the limit of clipping). NAb titer ≥ 4.32 log_2_ was recognized as a criterion for sufficient vaccine immunogenicity.

### Cytokines Assessment

The cytokines IL-1β, IL-12, and IFN-*ɣ* were measured in the BALB/c mice sera by means ELISA kits (Cusabio, WuhanHuamei, and BiotechCo. Ltd, China) in accordance with the manufacturer’s protocol. The results were presented in pg/ml.

### Statistical Analysis

All parameters were recorded for individual mouse of each group. Statistical data comparisons and plotting were done using version 8.3.1 of GraphPad Prism software (La Jolla, CA, United States). The statistical significance of the differences was determined using a one-way ANOVA with the multiple Tukey’s and Dunnett’s tests: ns = not significant, *p < 0.05, **p < 0.01, ***p < 0.005, ****p < 0.0001. P value < 0.05 was considered statistically significant. The results were presented as the average geometric values of the NAb in binary logarithms. Quantitative evaluation of cytokines was performed using the CurveExpert software. All experiments were accomplished independently at least three times.

## Results

### Evaluation of Humoral Immune Response

**Figure 1:**
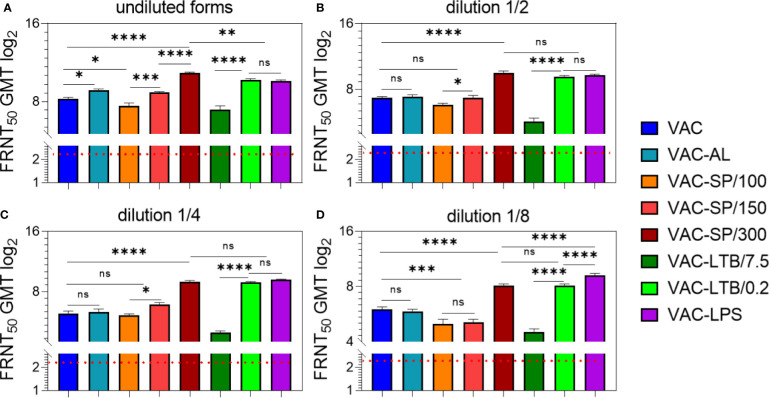
Antibody responses to experimental vaccines after BALB/c two immunizations with experimental vaccines and controls. Blood sera samples were collected from the mice (n = 7 for each group) 2 weeks after second i/m immunizations: **(A)** undiluted; **(B)** in dilution ½; **(C)** in dilution ^1^/_4_; **(D)** in dilution ^1^/_8_. Groups of seven mice were immunized with: VAC; VAC-AL; VAC-SP/100; VAC-SP/150; VAC-SP/300; VAC-LTB/7.5; VAC-LTB/0.2; VAC-LPS. The mean values and standard deviations for each group of seven mice were given in the graph. The FRNT_50_ limit of detection was a titer of control group < 2.32 log2. ns = not significant, *p < 0.05, **p < 0.01, ***p < 0.005, ****p < 0.0001 using a one-sided ANOVA with Tukey’s multiple comparisons test.

NAbs were detected in all experimental groups in 7/7 immunized mice up to ^1^/_8_ vaccines dilution. NAbs were absent in the blood sera of mice prior to immunization, as well as in the control groups. Evaluation of adjuvants’ effect on vaccine immunogenicity was carried out after two and three immunizations. Statistically significant differences in the geometric mean titer (GMT) of NAb between II and III immunizations were revealed only in the group with AL, where the NAb decreased after the third immunization and in the group LTB/0.2, where the NAb increased after the third immunization, compared with the second immunization. After the second and third immunizations with other vaccines, the NAb GMT had no statistically significant differences (**Figure 1**; **Supplementary Figure 1**).

### Immunization With Undiluted Vaccines

After the second immunization with VAC-AL, the NAb GMT was higher than after VAC immunization (p < 0.01) (**Figure 1A**). In groups immunized with VAC-LTB/7.5 and VAC-SP/100 Nab, GMT didn’t differ significantly and were lower than in the group immunized with VAC (p < 0.01) and (p < 0.05), respectively.

NAb GMT was significantly higher in the groups immunized with vaccines supplemented with LTB/0.2, LPS, SP/150 and SP/300 than in the VAC (p < 0.0001), (p < 0.0001), (p < 0.05) and (p < 0.0001), respectively (**Figure 1A**). There were no statistically significant differences between groups immunized with VAC-LTB/0.2 and VAC-LPS; however, NAb level in these groups was statistically lower than in the VAC-SP/300 group (p < 0.01). At the same time, NAb GMT in the VAC-SP/100 was significantly lower than in those immunized with the VAC-SP/150 (p < 0.005) and VAC-SP/300 (p < 0.0001), respectively.

After the third immunization with the experimental vaccines VAC-LTB/0.2, VAC-LPS, VAC-SP/150 and VAC-SP/300 immune response was the same as after second immunization, wherein the NAb GMT of mice immunized with VAC-SP/300 was higher than in the other groups. These three adjuvants significantly enhance the humoral immune response compared with VAC (p < 0.0001) (**Supplementary Figure 1A**).

### Immunization With the Vaccines in Dilutions of 1/2 and 1/4

There were no significant differences of the NAb GMT in mice sera in response to VAC-AL, VAC-SP/100, VAC-SP/150 immunizations in comparison to VAC (**Figures 1B, C**). NAb GMT in the VAC-SP/100 was significantly lower than in those immunized with the VAC-SP/150 (p < 0.05) and VAC-SP/300 (p < 0.0001), respectively. At the same time after the second immunization NAb GMT in the VAC-LTB/7.5 was significantly lower (p < 0.0001) than in those immunized with the VAC and VAC-LTB/0.2 (**Figures 1B, C**). There were also no significant differences between the VAC-SP/300, VAC-LTB/0.2 and VAC-LPS groups, and NAb in these groups were higher than in the VAC (p < 0.0001) (**Figures 1B, C**).

After the third immunization with VAC-AL, the NAb GMT was significantly lower than after the second immunization (p < 0.05) and lower than in VAC (p < 0.05) (**Supplementary Figure 1B, C**). There was no significant difference in NAb between the mice immunized with VAC-SP/100, VAC-SP/150 and those immunized with the VAC. The VAC-LTB/7.5 group retained a lower NAb compared with VAC (p < 0.0001). After the second and third immunizations, there were no statistically significant differences in NAb GMT in the groups VAC-LTB/0.2, VAC-LPS, and VAC-SP/300.

### Immunization With the Vaccines in Dilution of 1/8

There were no significant differences in NAb GMT in response to VAC and VAC-AL, VAC-SP/100, and VAC-SP/150. However, the NAb GMT was significantly higher in the group immunized with VAC compared to the group immunized with VAC-SP/100 (p < 0.005), VAC-SP/150 (p < 0.005) and VAC-LTB/7.5 (p < 0.0001). The NAb GMT after VAC-SP/300 and VAC-LTB/0.2 immunization didn’t differ significantly, and was lower than the NAb GMT in the group immunized with VAC-LPS (p < 0.0001) (**Figure 1D**). The similar dynamic of neutralizing antibodies was observed after the third immunizations (**Supplementary Figure 1D**).

**Figure 2:**
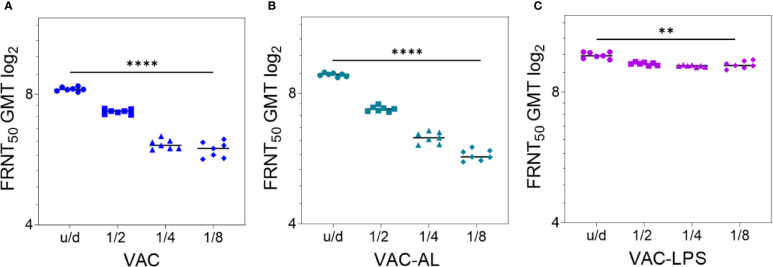
Correlation of NAb titer with antigenic load after two i/m immunizations with the experimental vaccines undiluted and in dilutions 1/2; 1/4; 1/8. Serum samples (n = 7 for each group) were collected from the mice 2 weeks after the second immunization. Groups of mice were immunized with: **(A)** VAC; **(B)** VAC-AL; **(C)** VAC-LPS. Sera were tested in FRNT_50_. NAb titers for individual mice are shown. The FRNT_50_ limit of detection was a titer of control group < 2.32 log2. **p < 0.01, ****p < 0.0001 using a one-sided ANOVA with Tukey’s multiple comparisons test.

A dose-dependent effect was observed in all groups (p < 0.0001) (**Figure 2** **;** **Supplementary Figure 2**). NAb GMT in the group VAC-AL increased only in u/d form in comparison with the group VAC (p < 0.01). However, in subsequent dilutions, the NAb GMT did not differ or was lower in the VAC-AL (**Figures 2A, B**). SPs—300 μg/ml, LTB—0.2 μg/ml (**Supplementary Figures 2C, E**) and LPS—50 μg/ml (**Figure 2С**) resulted in a less pronounced decrease in the NAb GMT depending on the dilutions. Decrease of spherical particle concentration (VAC-SP/100) declines the VAC immunogenicity (**Supplementary Figure 2A**). In the group VAC-LTB/7.5 the adjuvant load at a concentration of 7.5 had an immunosuppressive effect on the NAb production. The decrease in the NAb GMT was statistically significant and depended on the dilution of the vaccine (p < 0.0001) (**Supplementary Figure 2D**).

Thus, the most augmenting immune response was observed in the groups vaccinated with VAC-LPS, VAC-SP/300 and VAC-LTB/0.2 (p < 0.0001). It is important to emphasize that the immunostimulating effect was more pronounced in dilutions of these vaccines.

In subsequent experiments, VAC, VAC-AL, and VAC-LPS were studied to determine their stability.

### Effect of Adjuvants on the Vaccines Immunogenicity During Storage

Vaccines VAC, VAC-LPS were stored at 6 ± 2°C in liquid and lyophilized forms, and VAC-AL in liquid form. Control for immunogenic stability was carried out after 12 months of storage in regulated conditions. Experimental vaccines were administered in u/d form and in a dilution of 1/8. Immunogenicity control was conducted after the second immunization.

**Figure 3:**
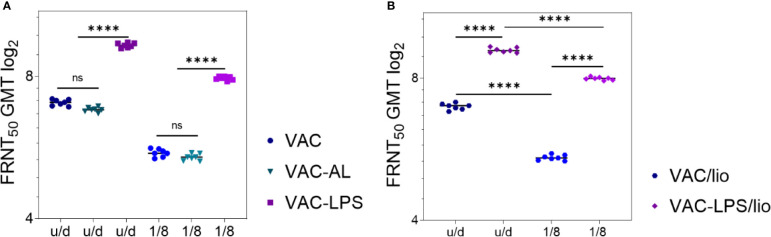
Adjuvants’ influence on the immunogenicity/stability of the experimental vaccines during storage for 12 months. NAb titers after two immunizations with vaccines undiluted and in 1/8 dilution: **(A)** Liquid form; **(B)** Lyophilized form. Serum samples were collected (n = 7 for each group) 2 weeks after the second immunization. The GMT for each group of seven mice was given in the graph. The FRNT_50_ limit of detection was a titer of control group < 2.32 log2. Statistical analysis was performed using a one-sided ANOVA with Tukey’s multiple comparisons test: ns = not significant, ****p < 0.0001.

NAbs were detected in 7/7 immunized mice of all experimental groups up to ^1^/_8_ vaccine dilution. NAbs were not detected in the blood serum of mice prior to immunization, as well as in the control groups. After immunization with undiluted vaccines stored in liquid form for 12 months NAb GMT decreased in the VAC (p = 0.01) and VAC-AL (p < 0.01) groups (**Figure 3A**). However, the NAb GMT of mice immunized with VAC-LPS was significantly higher (p < 0.0001) in comparison with VAC and are not statistically different from the initial NAb GMT (**Figure 3A**). In vaccines dilution ^1^/_8_ the NAb titers significantly decreased (p < 0.0001) both in liquid and lyophilized forms of vaccines, except of VAC-LPS (**Figures 3A, B**). There were no significant differences in the immunogenicity of vaccines stored in both liquid and lyophilized forms (**Figures 3A, B**).

### Cytokine Evaluation

Based on the specific roles of IL-12 and IFN-*ɣ* in adaptive immune response, we hypothesized that inactivated PUUV vaccine would stimulate their expression in mice blood.

The regulatory cytokines IL-12, IFN-*ɣ*, and proinflammatory IL-1β in BALB/c mice blood sera prior to the first (PI) and after second immunizations have been defined. The background cytokine level was assumed to be the level of cytokines in the PI. Evaluation of the adjuvants’ immunomodulating effect was carried out by increasing the cytokines in the mice blood sera after immunizations with VAC, VAC-AL, VAC-LPS, VAC-SP/100, VAC-SP/300, and VAC-LTB/7.5, VAC-LTB/0.2. Control groups included: PI, C, C-AL, C-LPS, C-SP/100, C-SP/300, C-TLB/7.5, C-LTB/0.2 (**Supplementary Figure 3**).

**Figure 4:**
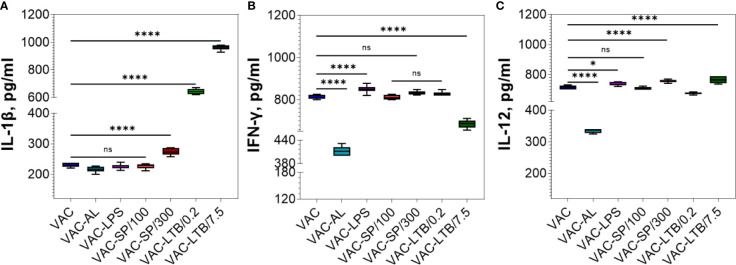
Comparative analysis of the cytokine profile in BALB/c mice sera after two immunizations. Cytokines were detected by means of commercial ELISA kits. IL-1β **(A)**, IFN-*ɣ* **(B)** and IL-12 **(C)** levels in the experimental groups. The mean values and standard deviations for each group of seven mice were given in the graph. ns = not significant, *p < 0.05, ****p < 0.0001 using a one-sided ANOVA with Dunnett’s multiple comparisons test.

IL-1β significantly increased in the C-LTB/0.2, C-LTB/7.5 and to a much lesser extent in the SP/300 (p < 0.0001) (**Supplementary Figure 3A**). As in the control groups, IL-1β was increased in VAC-LTB/0.2 and VAC-LTB/7.5 and significantly less in VAC-SP/300 (p < 0.0001) group (**Figure 4A**). In contrast, there were no essential responses in the other groups (**Figure 4A**; **Supplementary Figure 3A**).

IFN-*ɣ* increased in the C-SP/300, C-LTB/0.2 and C-LTB/7.5 as compared with PI (p < 0.0001) (**Supplementary Figure 3B**) and VAC-LPS, VAC-SP/300 over against VAC (**Figure 4B**). Less pronounced rise of this cytokine was observed in the VAC-AL and VAC-SP/100, VAC-LTB/7.5 groups (p < 0.0001) (**Figure 4B**). There was no significant difference in IFN-*ɣ* after immunization with VAC-SP/100, VAC-LTB/0.2 and VAC (**Figure 4B**).

IL-12 also increased in the immunized mice blood sera with all the experimental vaccines (**Figure 4C** **;** **Supplementary Figure 3A**).

At 14 day post second immunization the cytokine level increased prominently compared to PI. The cytokine response after two and three immunizations had no quantitative differences (data not shown). There were no statistically significant differences in cytokine profile after the second immunization with candidate vaccines stored for 12 months in liquid and lyophilized forms (data not shown).

## Discussion

We investigated the efficacy of four adjuvants of various classes in the composition with Puumala virus vaccine. The known adjuvants are able to improve inactivated vaccines: reduce the dose of antigen, accelerate a pronounced and prolonged immune response. Due to the wide variety of adjuvants and modes of action, their selection is carried out by comparing their immune effectiveness, as well as evaluating their safety and tolerability in animal models.

Aluminum sorbed vaccine showed the same NAb immune response as vaccine alone. The smallest increase in IFN-*ɣ* and IL-12 cytokines compared with other adjuvants was also noted (**Figure 4**).

These results are in line with data of other authors on aluminum salts’ poor immunostimulation activity, especially for cellular immune response (McKee and Marrack, 2017).

The next candidate to improve PUUV vaccine was a promising plant virus-based adjuvant, SP, derived by thermal transition of Tobacco mosaic virus. In previous studies it was demonstrated that SPs could potentiate immune response to proteins and virions (Karpova et al., 2012; Trifonova et al., 2014; Trifonova et al., 2017). In our experiments, an augmenting immune response was observed at 150 μg/ml SPs at least. SPs’ maximal adjuvant effect was at the concentration of 300 μg/ml and was similar to LPS and LTB/0.2. Despite the noticeable concentration of SP per dose, it is worth noting that SPs showed good safety in toxicity studies in different animal models with comparable and large quantities (Nikitin et al., 2018a). Here we have shown the first time that SPs have adjuvant activities for IL-12 and IFN-*γ* cytokine production.

LTB administration to BALB/c mice at the concentration of 7.5 μg/ml both alone and as part of the vaccine was accompanied by a pronounced pro-inflammatory response, which expressed a significant increase of the IL-1β level. Interleukin-1β plays an important role in early immune responses, and dysregulation of its activity leads to auto-inflammatory (autoimmune) manifestations (Maedler et al., 2002; Fillman et al., 2013; Veerdonk and Netea, 2013; Palomo et al., 2015; Bent et al., 2018); therefore, excessive induction of IL-1β during immunization with vaccines is undesirable. LTB at the concentration 0.2 μg/ml induced IL-1β in a smaller amount, while the level of IFN-*ɣ* found in the mice sera did not differ much from the values detected in groups VAC-LPS and VAC-SP/300.

Novel clinically applicable apyrogenic low-endotoxic LPS from *Shigella sonnei* resulted in both a high NAbs level in mice sera and also preserve NAbs after a one-year storage of the vaccine. LPS did not cause an increase in pro-inflammatory IL-1β and contributed to a significant increase in IFN-*ɣ* and IL-12.

Cytokine analysis clearly demonstrated that all of the experimental vaccines evoke significant increase in IFN-*γ* and IL-12. It is known that the induction of regulatory cytokines IFN-*γ* and IL-12 polarizes the immune response according to the Th-1 type (Zhu et al., 2009). Interleukin-12 provides a regulatory pathway through interactions of cells with antigens and directs specific immunity according to the corresponding T-cell phenotype (Hsieh et al., 1993).

According to our data, experimental vaccines with the exception of VAC-SP/300, VAC-LTB/0.2, and VAC-LPS, evoke a dose-dependent immune response. VAC-SP/300, VAC-LTB/0.2, and VAC-LPS maintained the initial level of NAbs up to the vaccine dilution of 1/8 which allows the antigenic load in the vaccine to be reduced by at least eight times. To determine the possibility of further reducing the antigenic load, it is planned to conduct studies in the future with these adjuvants, including dilution of the vaccine up to 1/256 with immunogenicity control after the first and second immunizations.

NAbs decreased in the VAC, VAC/AL unlike VAC/LPS when stored at 6 ± 2°C for 1 year. The form of the vaccine (liquid or lyophilized) does not affect immunogenicity for at least 1 year storage at 6 ± 2°C. It was found that neutralizing antibodies and the cytokines in the mice blood sera did not increased statistically significant after the third immunization compared with two immunizations. These results make it possible to recommend twofold immunization for future preclinical and clinical trials of hantavirus vaccines.

Summarizing our results, one can conclude that aluminum hydroxide, which is rather widely used as an adjuvant for inactivated vaccines, does not enhance the humoral response, nor does it stabilize PUUV vaccine during storage under regulated conditions. Fairly optimistic results on enhancing the immune response to the PUUV vaccine were obtained for thermally denatured Tobacco mosaic virus, SPs, at a concentration of 300 μg/ml; LTB, a thermolabile enterotoxin B, at a concentration of 0.2 μg/ml; LPS, *S. Sonnei* highly homogenous S-LPS with tri-acylated lipid A moiety in a dose of 50 μg/ml. In the presence of these adjuvants, a dose-dependent, statistically significant increase in the NAb titer was observed, as well as regulatory cytokines IL-12 and IFN-*ɣ*. The effect on the vaccine stability during 1 year storage was studied for LPS, and these results showed that even if the vaccine dose was reduced by eight times, the Nab titer did not decrease. At the same time, the immune response to the native vaccine and the vaccine supplemented with aluminum hydroxide decreased markedly, which was especially noticeable when the vaccine dose was eight times reduced.

It can be assumed, that LPS in HFRS vaccine formulation is promising to enhance the immune response, reduce antigenic load, and stabilize vaccine during storage. In accordance with previous clinical trials, LPS is safe for parenteral administration for humans in the dose we used (Ledov et al., 2019), slightly increases the protein load in the vaccine (50 μg/ml) and can be recommended in hantavirus vaccine formulation. The main findings of this study predominantly related to hantavirus vaccines, but can probably be extrapolated to other inactivated vaccines which require experimental affirmation.

## Data Availability Statement

The raw data supporting the conclusions of this article will be made available by the authors, without undue reservation.

## Ethics Statement

The animal study was reviewed and approved by Chumakov Federal Scientific Center for Research and Development of Immune-and-Biological Products of Russian Academy of Sciences.

## Author Contributions

ET and TD developed the original idea. ET, TD, and SK designed the whole study. AI solved organizational issues. NN, EE, OK, AM, PA, and VL’V provided adjuvants for the study. TD, SK, ME, and MB constructed the experimental vaccines. SK and ME performed the experiments, evaluated the immune responses, and took care of the animals. SK processed the data and calculated the statistics. SK, ME, TD, and PT drafted the manuscript. All authors contributed to the article and approved the submitted version.

## Conflict of Interest

The authors declare that the research was conducted in the absence of any commercial or financial relationships that could be construed as a potential conflict of interest.

## Footnotes

1
https://findpatent.ru/patent/228/2285539.html

## Supplementary Material

Supplementary Figure 1 Antibody responses to experimental vaccines after BALB/c three immunizations. Blood sera samples from the mice (n = 7 for each group) were collected 2 weeks after the last i/m immunizations: **(A)** undiluted; **(B)** in dilution ½; **(C)** in dilution ^1^/_4_; **(D)** in dilution ^1^/_8_. Groups of seven mice were immunized with: VAC; VAC-AL; VAC-SP/100; VAC-SP/150; VAC-SP/300; VAC-LTB/7.5; VAC-LTB/0.2; VAC-LPS. The mean values and standard deviations for each group of seven mice were given in the graph. The FRNT_50_ limit of detection is a titer of control group (< 2.32 log2). ns = not significant, *p < 0.05, **p < 0.01, ***p < 0.005, ****p < 0.0001 using a one-sided ANOVA with Tukey’s multiple comparisons test.

Supplementary Figure 2 Correlation of NAb titer with antigen load after two immunizations with the experimental vaccines undiluted and in dilutions 1/2; 1/4; 1/8. Blood sera were collected from the mice (n = 7 for each group) 2 weeks after second immunization. Groups of seven mice were immunized with: **(A)** VAC-SP/100; **(B)** VAC-SP/150; **(C)** VAC-SP/300; **(D)** VAC-LTB/7.5; **(E)** VAC-LTB/0.2. Sera were tested in FRNT_50_. NAb titers for individual mice are shown. The FRNT_50_ limit of detection was a titer of control group < 2.32 log2. **p < 0.01, ****p < 0.0001 using a one-sided ANOVA with Tukey’s multiple comparisons test.

Supplementary Figure 3 Comparative analysis of the cytokine profile in BALB/c mice sera after two immunizations. Cytokines were detected by means of commercial ELISA kits. Mice sera were taken the day prior to immunizations—pre-immune reference sera (PI), which were used as a negative control. IL-1β **(A)**, IFN-*ɣ* **(B)** and IL-12 **(C)** levels in the control groups. The mean values and standard deviations for each group of seven mice were given in the graph. ns = not significant, *p < 0.05, ***p < 0.005, ****p < 0.0001 using a one-sided ANOVA with Dunnett’s multiple comparisons test.
