# Supplementary figures and images for: Various Adjuvants Effect on Immunogenicity of Puumala Virus Vaccine
Source: Front Cell Infect Microbiol. 2020 Oct 26;10:545371. doi: 10.3389/fcimb.2020.545371 (PMC7649337; doi:10.3389/fcimb.2020.545371)

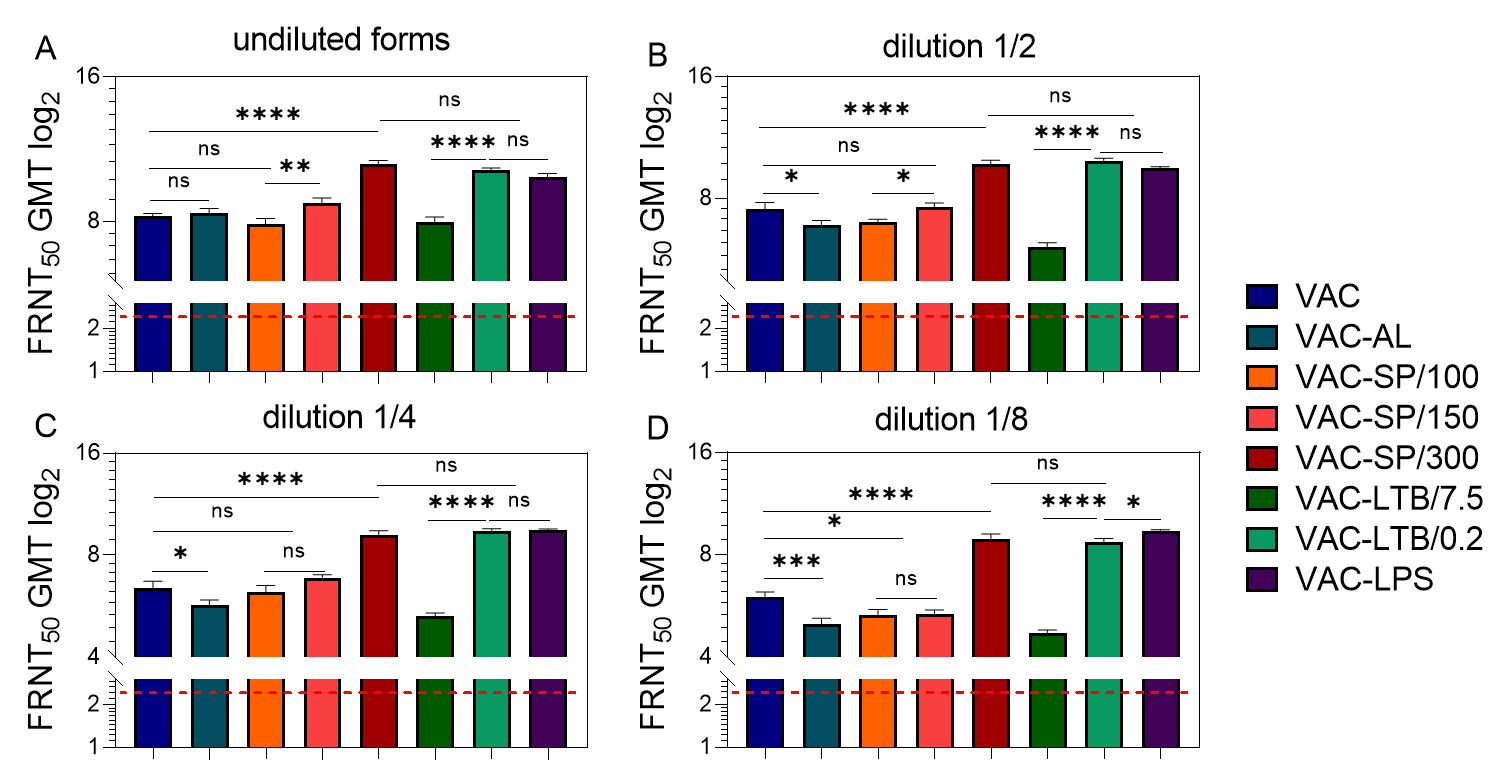

Supplement: Supplementary Figure 1 — Antibody responses to experimental vaccines after BALB/c three immunizations. Blood sera samples from the mice (n = 7 for each group) were collected 2 weeks after the last i/m immunizations: (A) undiluted; (B) in dilution ½; (C) in dilution 1/4; (D) in dilution 1/8. Groups of seven mice were immunized with: VAC; VAC-AL; VAC-SP/100; VAC-SP/150; VAC-SP/300; VAC-LTB/7.5; VAC-LTB/0.2; VAC-LPS. The mean values and standard deviations for each group of seven mice were given in the graph. The FRNT50 limit of detection is a titer of control group (< 2.32 log2). ns = not significant, *p < 0.05, **p < 0.01, ***p < 0.005, ****p < 0.0001 using a one-sided ANOVA with Tukey’s multiple comparisons test. [file Image_1.tiff]

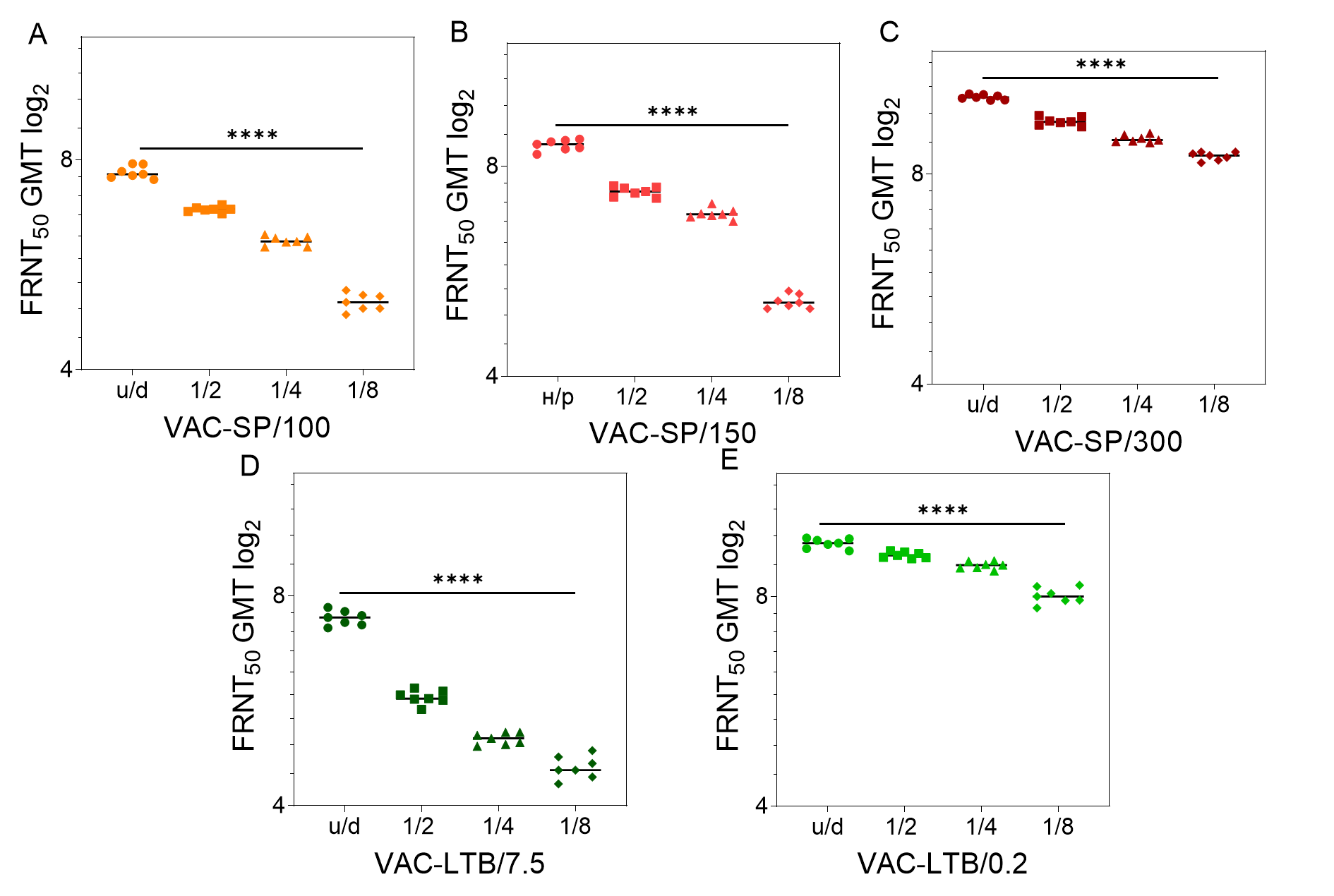

Supplement: Supplementary Figure 2 — Correlation of NAb titer with antigen load after two immunizations with the experimental vaccines undiluted and in dilutions 1/2; 1/4; 1/8. Blood sera were collected from the mice (n = 7 for each group) 2 weeks after second immunization. Groups of seven mice were immunized with: (A) VAC-SP/100; (B) VAC-SP/150; (C) VAC-SP/300; (D) VAC-LTB/7.5; (E) VAC-LTB/0.2. Sera were tested in FRNT50. NAb titers for individual mice are shown. The FRNT50 limit of detection was a titer of control group < 2.32 log2. **p < 0.01, ****p < 0.0001 using a one-sided ANOVA with Tukey’s multiple comparisons test. [file Image_2.tiff]

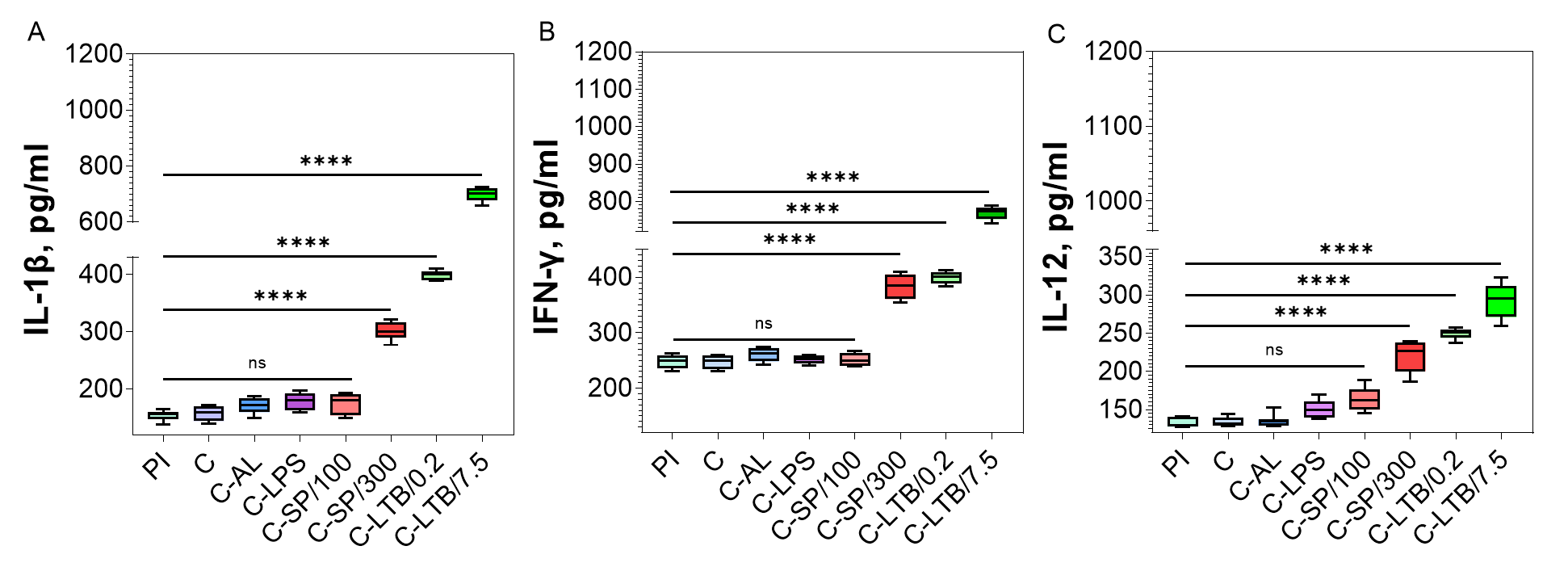

Supplement: Supplementary Figure 3 — Comparative analysis of the cytokine profile in BALB/c mice sera after two immunizations. Cytokines were detected by means of commercial ELISA kits. Mice sera were taken the day prior to immunizations—pre-immune reference sera (PI), which were used as a negative control. IL-1β (A), IFN-ɣ (B) and IL-12 (C) levels in the control groups. The mean values and standard deviations for each group of seven mice were given in the graph. ns = not significant, *p < 0.05, ***p < 0.005, ****p < 0.0001 using a one-sided ANOVA with Dunnett’s multiple comparisons test. [file Image_3.tiff]
